# Hyperbaric Oxygen Reduces Production of Reactive Oxygen Species in Neutrophils from Polytraumatized Patients Yielding in the Inhibition of p38 MAP Kinase and Downstream Pathways

**DOI:** 10.1371/journal.pone.0161343

**Published:** 2016-08-16

**Authors:** Deborah Grimberg-Peters, Carina Büren, Joachim Windolf, Thorsten Wahlers, Adnana Paunel-Görgülü

**Affiliations:** 1 University Hospital Düsseldorf, Department of Trauma and Hand Surgery, Moorenstrasse 5, 40225, Düsseldorf, Germany; 2 Heart Center of the University of Cologne, Department of Cardiothoracic Surgery, Kerpener Str. 62, 50937, Cologne, Germany; Klinikum rechts der Isar der Technischen Universitat Munchen, GERMANY

## Abstract

Trauma represents the leading cause of death among young people in western countries. Among the beneficial role of neutrophils in host defence, excessive priming and activation of neutrophils after major trauma lead to an overwhelming inflammatory response and secondary host tissue injury due to the release of toxic metabolites and enzymes. Hyperbaric oxygen (HBO) therapy has been proposed to possess antiinflammatory effects and might represent an appropriate therapeutic option to lower inflammation in a broad range of patients. Here, we studied the effects of HBO on the activity of neutrophils isolated from severely injured patients (days 1–2 after trauma), in fact on the production of reactive oxygen species (ROS) and release of neutrophil extracellular traps (NETs). We found exposure to HBO therapy to significantly diminish phorbol-12-myristate-13-acetate (PMA)-induced ROS production in neutrophils isolated from patients and healthy volunteers. At the same time, marked decrease in NETs release was found in control cells and a less pronounced reduction in patient neutrophils. Impaired ability to produce ROS following exposure to HBO was demonstrated to be linked to a strong downregulation of the activity of p38 MAPK. Only slight suppression of ERK activity could be found. In addition, HBO did not influence neutrophil chemotaxis or apoptosis, respectively. Collectively, this study shows for the first time that HBO therapy suppresses ROS production in inflammatory human neutrophils, and thus might impair ROS-dependent pathways, e.g. kinases activation and NETs release. Thus, HBO might represent a feasible therapy for patients suffering from systemic inflammation, including those with multiple trauma.

## Introduction

Severe inflammation induced by major trauma and ischemia-reperfusion injury increases the risk of development complications such as sepsis, acute respiratory distress syndrome (ARDS) and organ failure [1]. Neutrophils, which comprises up to 70% of total circulating leukocytes in humans, are critically involved in the inflammatory response to injury. During infection or sterile inflammation, activated neutrophils accumulate in the damaged tissue or organ and release a number of chemokines and cytokines, such as IL-8, IL-6, IL-1ß and TNF-α [2–4]. These mediators may participate in recruiting more neutrophils and subsequent massive release of reactive oxygen species (ROS) and proteases promote tissue injury and endothelial dysfunction [5]. Neutrophils can also counteract invading pathogens by directly phagocytosing microbes or releasing cytotoxic molecules by degranulation, including ROS, proteases, collagenases, and myeloperoxidase (MPO) [6]. In sterile inflammation, such as trauma, neutrophils are known to produce elevated levels of spontaneous ROS and uncontrolled release of aggressive oxygen molecules contributes to vascular leakage, predisposing patients to organ injury. Indeed, trauma patients developing ARDS display higher levels of ROS production when compared to control trauma patients [7]. Aside from phagocytosis and degranulation, the release of NETs during inflammation and infection has received much attention during the last years. They are formed in response to a variety of pro-inflammatory stimuli, such as IL-8 and LPS [8], activated platelets [9], activated endothelial cells [10] and consist of decondensed chromatin and antimicrobial proteins. During formation of NETs, neutrophils usually die and this process is called NETosis. However, recently it became evident that NETs might be release by vital neutrophils, also the mechanism is somehow different from the classical NETosis [11]. NETs release was shown to be dependent on the production of ROS via NADPH-oxidase 2 (NOX-2), the migration of the protease neutrophil elastase (NE) and myeloperoxidase (MPO) from granules to the nucleus as well as on histone citrullination catalysed by peptidylarginine deiminase (PAD4) [12]; [13, 14]. The requirement for ROS production is consistent with the findings that neutrophils treated with the pharmacological NADPH-oxidase inhibitor diphenyleneiodonium (DPI) and patients with defective NADPH-oxidase are unable to release NETs [15]. However, apart from their antimicrobial function, uncontrolled NETs release following, sepsis, trauma and ischemia-reperfusion injury can contribute to tissue destruction by damaging epithelial and endothelial cells [16]. Previous studies have reported that NETs may predict posttraumatic complications in the intensive care setting and NETs levels have also been demonstrated to be elevated in trauma patients who subsequently developed sepsis [17, 18].

Hyperbaric oxygen (HBO) therapy was already demonstrated to reduce inflammation and is widely recognized to be an effective treatment for chronic wounds. As demonstrated in an experimental setting using a rat skin wound healing model as well as in healthy human subjects, HBO modulates cytokine release, increases ROS production, reduces apoptosis and modulates leukocyte activation and adhesion [19, 20]. As HBO has been found to inhibit the adhesion of neutrophils to the endothelium following trauma, it has been proposed as an adjunctive therapy. Several studies using animal models indicated that neutrophil recruitment is significantly reduced by HBO following ischemia / reperfusion injury to the different organs [21, 22]. Additionally, reduced tissue necrosis and lipid peroxidation have been reported [23, 24].

In this study, we aimed to elaborate the effect of hyperbaric oxygen treatment on the activity of neutrophils from critically ill patients with respect to their ability to produce ROS and to release NETs.

## Materials and Methods

### Study population

Study approval was obtained from the local ethics committee of the University of Düsseldorf, Germany (# 4947). Written informed consent was obtained from all patients or their legal representatives, respectively. Ten patients with blunt or penetrating multiple injuries who were admitted to our Level I Trauma Center with an Injury Severity Score (ISS) > 16, systemic inflammatory response syndrome (SIRS), intensive care unit (ICU) stay > 3 days and aged 18 years and older were enrolled in this study. Patients with known preexisting immunological disorders or systemic immunosuppressive medication, an ISS < 16 or severe traumatic brain injuries (head AIS ≥ 3) were excluded. The latter group has been shown to exhibit an altered inflammatory response as compared to polytraumatized patients without traumatic brain injury [25, 26]. The severity of injury was assessed by the ISS, based on the abbreviated injury scale (AIS; [27]). In fact, taking into account to the AIS, head injuries occured in 60% of cases, 50% of patients suffered from face injuries, 90% from thoracal injuries, 100% from abdominal injuries, 80% from pelvic or extremital injuries and 50% from external injuries, like e.g. hypothermia. SIRS was defined using the criteria outlined 2005 by the International Sepsis Forum [28]. SIRS was defined by two or more of the following criteria: temperature > 38°C or < 36°C; heart rate > 90 beats per minute; respiratory rate > 20 breaths per minute or arterial carbon dioxide tension (PaCO_2_) < 32 mmHg; and white blood cell count > 12.000 cells/mm^3^ or < 4.000 cells/mm^3^, or with > 10% immature (band) forms. Heparinized blood (10 ml) was collected from healthy volunteers and from trauma patients during the first 48 h after admission to the trauma center. Blood samples were immediately used for neutrophil isolation.

### Isolation of human neutrophils

Neutrophils were isolated from severely injured patients and healthy volunteers, respectively, by density gradient centrifugation on percoll (Millipore). The method has previously been described in detail [29]. Isolated PMN were suspended in RPMI 1640, supplemented with 2 mM glutamine, 100 U/ml penicillin, 100 μg/ml streptomycin and 5% or 10% FCS, respectively. Purity and viability were routinely above 95% as assessed by flow cytometry.

### Hyperbaric treatment

Freshly isolated neutrophils (2x10^6^/ml) were seeded into 48 well plates and further placed into a HBO chamber (HAUX TESTCOM 200/2 chamber) at 95% O2 and 2.0 atmosphere (atm) for 120 min. Control cells were incubated under normobaric conditions for the same time.

### Quantification of NETs

Neutrophils were stimulated with phorbol-12-myristate-13-acetate (PMA, 20 nM) for 3 h at 37°C in a humidified atmosphere containing 5% CO_2_. NETs quantification in culture supernatants was performed using the Quant-iT Pico Green dsDNA assay as already described by our group [17, 18, 30] and by following the manufacturer’s instructions (Invitrogen GmbH, Darmstadt, Germany). The fluorescence intensity reflects the amounts of DNA and was measured at excitation and emission wavelengths of 485 nm and 530 nm, respectively in a microplate reader (Victor3, PerkinElmer, Waltham, USA). A standard calibration curve by means of defined calf thymus DNA (Sigma, St. Louis, USA) amounts ranging from 0 to 2 μg/ml has been used in all analyses.

### Detection of intracellular ROS

To quantify ROS production, the ROS-reactive dye DHR was used which diffuses in to the cells is converted to cationic, green fluorescent rhodamine 123 upon oxidation. Neutrophils (2x10^6^/ml) were treated with 20 μM DHR 123 and cells were further stimulated with 10 nM PMA for 10 min at 37°C. The reaction was stopped on ice and cells were centrifuged at 1500 rpm for 5 min. Then, cells were resuspended in ice-cold PBS and the fluorescence intensity was analysed immediately at excitation and emission wavelengths of 485 nm and 530 nm, respectively in a microplate reader (Victor3, PerkinElmer, Waltham, USA). The ROS concentration was expressed as relative fluorescence units (RFU).

### Chemotaxis Assay

Human neutrophil chemotaxis was measured in 24-well plates containing transwell inserts with 3 μm pore size (Transwell Permeable Supports, 3.0 μm Polycarbonate Membrane, Costar, Corning NY). Transwell inserts containing 100 μl (1 × 10^5^) of cells were placed in wells containing RPMI 1640, supplemented with 2 mM glutamine, 100 U/ml penicillin, 100 μg/ml streptomycin, 5% FCS and 25 ng/ml of IL-8 (R&D Systems) as chemoattractant. After incubation of plates for 30 min at 37°C, the transwell inserts were removed and fluorescent CountBright counting beads (Invitrogen) were added to samples to quantify absolute cell numbers by flow cytometry (FACSCalibur, BD).

### Western blot

Western blot analysis was performed as described previously [31]. In brief, cells were suspended in RIPA buffer supplemented with complete mini protease inhibitor cocktail and phosphatase inhibitor cocktail (both Roche) followed by sonication. Protein was separated on SDS-PAGE and transferred to nitrocellulose membranes. The following antibodies have been used: mouse monoclonal anti-human Mcl-1 (BD Pharmingen), mouse monoclonal anti-GAPDH (Imgenex), rabbit polyclonal anti-human p44/42 MAPK (Erk1/2), Phospho-p44/42 MAPK (Erk1/2) (Thr202/Tyr204), p38 MAPK, and Phospho-p38 MAPK (Thr180/Tyr182) (all Cell Signaling Technology). The membranes were further incubated with HRP-conjugated secondary Abs (goat anti-rabbit IgG, goat anti-mouse IgG; Dako). Protein expression was quantified by densitometry.

### Quantification of neutrophil apoptosis

Neutrophil apoptosis was quantified as previously described in detail [31].

### Statistical analyses

Statistical analyses were performed using GraphPad Prism 5.0 (GraphPad Software, San Diega, CA, USA). Data are presented as mean ± SEM. Data were tested using one-way ANOVA followed by Newman-Keuls post-test. Differences between data groups were considered to be statistically significant at p < 0.05.

## Results

### HBO reduces ROS production and NETs release from inflammatory neutrophils

**Fig 1:**
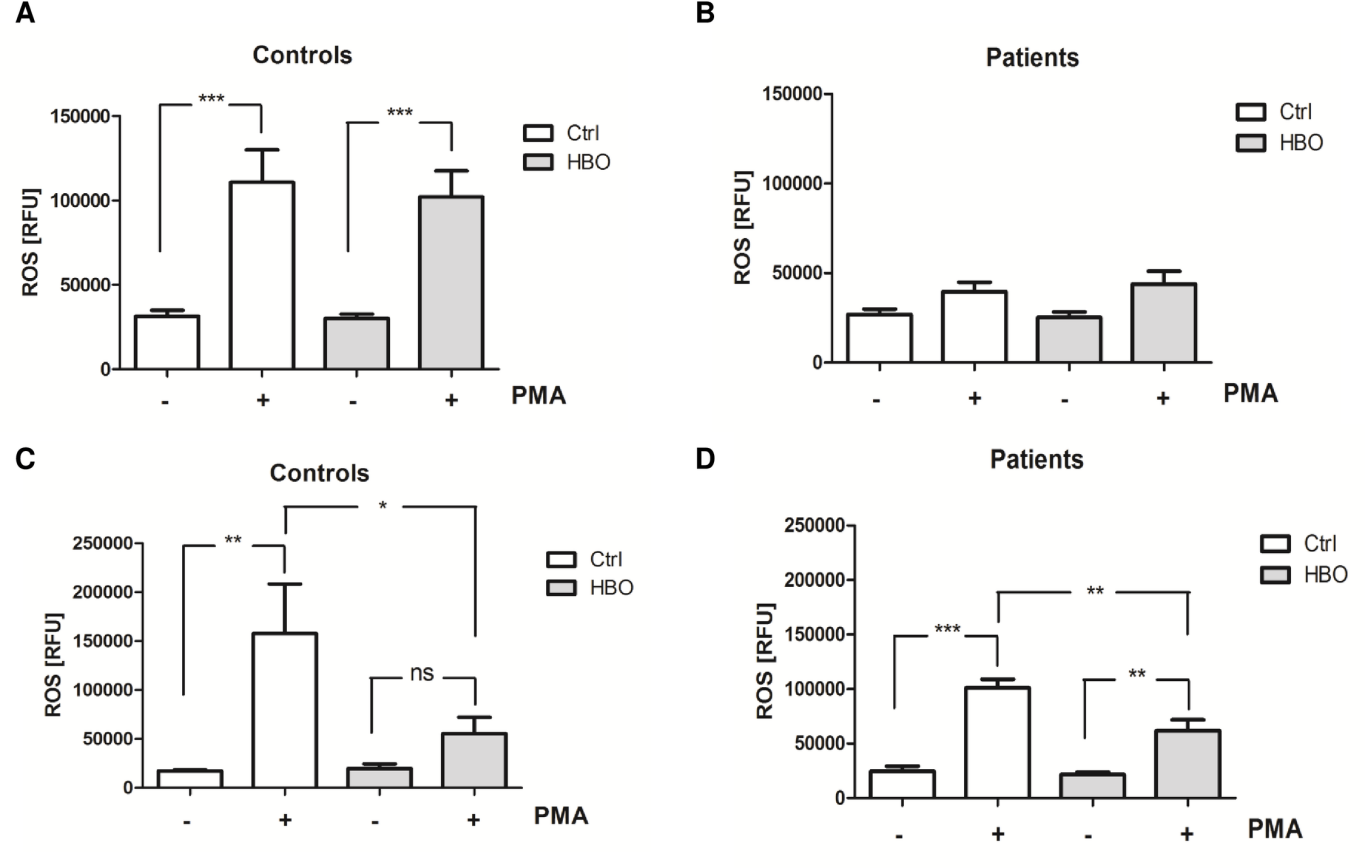
HBO impairs ROS production in control and inflammatory neutrophils. Freshly isolated neutrophils from healthy volunteers (Controls) (A, C; n = 6–11) and polytraumatized patients (Patients) (B, D; days 1–2 after trauma; n = 5–9) were exposed to HBO (HBO; 2.0 atm) for 120 min. Control cells (Ctrl) were incubated under normobaric, normoxic conditions for the same time. Cells were further stimulated with PMA (10 nM) for 10 min (A, B) and 3 hours (C, D), respectively. Intracellular ROS production was quantified by DHR 123 staining and expressed as relative fluorescence units (RFU). *p<0.05; **p<0.01; ***p<0.001; ns = not significant

Effective neutrophil activation is important for a successful host defense but in severe systemic inflammation elicited by trauma or sepsis, these cells contribute to collateral tissue damage during the initial inflammatory SIRS by releasing toxic molecules, including ROS. To test the effect of HBO on neutrophil oxidative burst, freshly isolated cells from polytraumatized patients (days 1–2 after trauma) and healthy volunteers were exposed to HBO at 2.0 bar for 120 min. As depicted in Fig 1, immediately upon HBO treatment, stimulation by PMA led to a marked increase in ROS production in control cells (Fig 1A) and to less pronounced raise in patient neutrophils (Fig 1B), without reaching significance. However, no effect of HBO on ROS production could be observed. We therefore aimed to evaluate the late effects of HBO treatment on neutrophil activity. To this end, PMA-stimulated neutrophils were further cultured for 3 h under cell culture conditions (21% O_2_, 5% CO_2_, 37°C) before ROS quantification. Indeed, ROS levels in neutrophils from volunteers (Fig 1C) and those isolated from polytraumatized patients (Fig 1D) exposed to HBO therapy were significantly diminished when compared to control cells, demonstrating that HBO may impede ROS production in primed cells.

**Fig 2:**
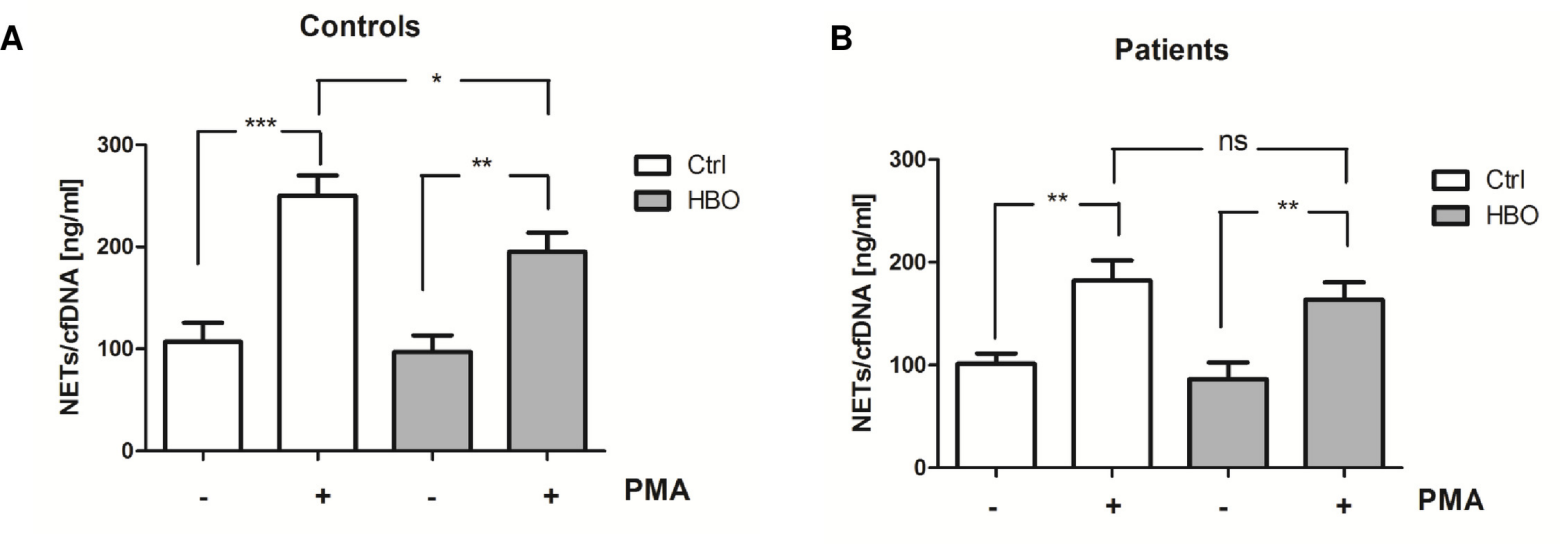
HBO reduces NETs release from neutrophils. Neutrophils from polytraumatized patients (Patients; n = 7) and healthy volunteers (Controls; n = 12) were exposed to HBO (HBO; 2.0 atm) for 120 min or cultured under normobaric and normoxic conditions (Ctrl). Then, cells were stimulated with PMA (20 nM) and incubated under cell culture conditions for 3 h. NETs / Cell-free DNA (cfDNA) in culture supernatants were quantified by Pico Green staining. *p<0.05; **p<0.01; ***p<0.001; ns = not significant

Taken into account, that ROS are critically involved in NETosis and neutrophils displaying mutations in the NADPH oxidase are unable to form NETs, we further quantified NETs levels. The release of NETs from human neutrophils upon PMA stimulation has previously been demonstrated by our group [18]. As displayed in Fig 2, neutrophils exposed to HBO were subsequently stimulated with PMA for 3 h and levels of NETs / cell free DNA (cfDNA) were detected in the cell supernatants. PMA led to a strong increase of NETs levels in both, neutrophils isolated from healthy volunteers and severely injured patients. Of note, HBO therapy significantly diminished the ability of control neutrophils to release NETs upon PMA stimulation (Fig 2A), whereas in trauma neutrophils only minor, not significant reduction of NETs could be observed (Fig 2B), despite strong suppression of ROS production found in these cells.

### HBO does not influence cell migration

**Fig 3:**
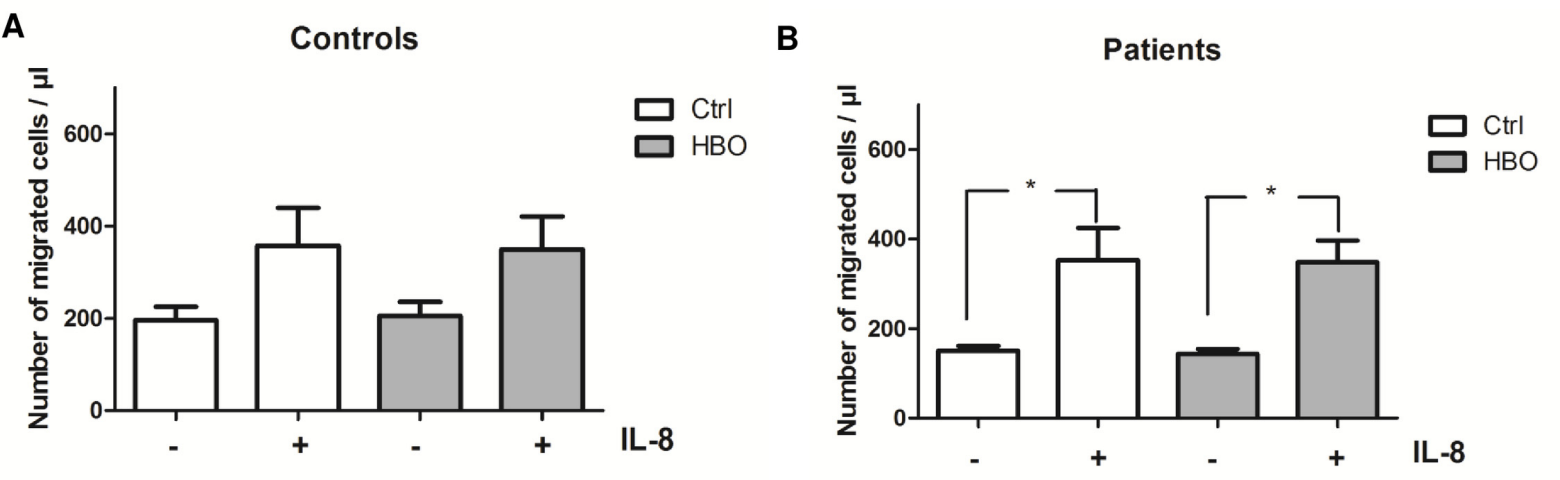
Effect of HBO on neutrophil chemotactic activity. After HBO (2.0 atm; 120 min), neutrophils from polytraumatized patients (Patients; n = 4) and volunteers (Controls; n = 6) were transferred into transwell inserts (3.0 μm pore size) and spontaneous as well as migration in response to IL-8 (25 ng/ml) were determined after 30 min of incubation under cell culture conditions. Cells incubated under normoxic and normobaric conditions served as controls. *p<0.05

It has been already described, that ROS might modulate neutrophil chemotaxis and cells treated with NADPH oxidase complex inhibitors display defective migration [32]. We therefore tested, if HBO influences cell migration in response to IL-8 (Fig 3). However, although significant increase in cell migration could only be detected after stimulation of trauma neutrophils with IL-8, HBO did not impair IL-8-mediated chemotactic activity of neutrophils, neither in those isolated from controls nor from patients.

### Effect of HBO on the activity of the kinases ERK and p38

**Fig 4:**
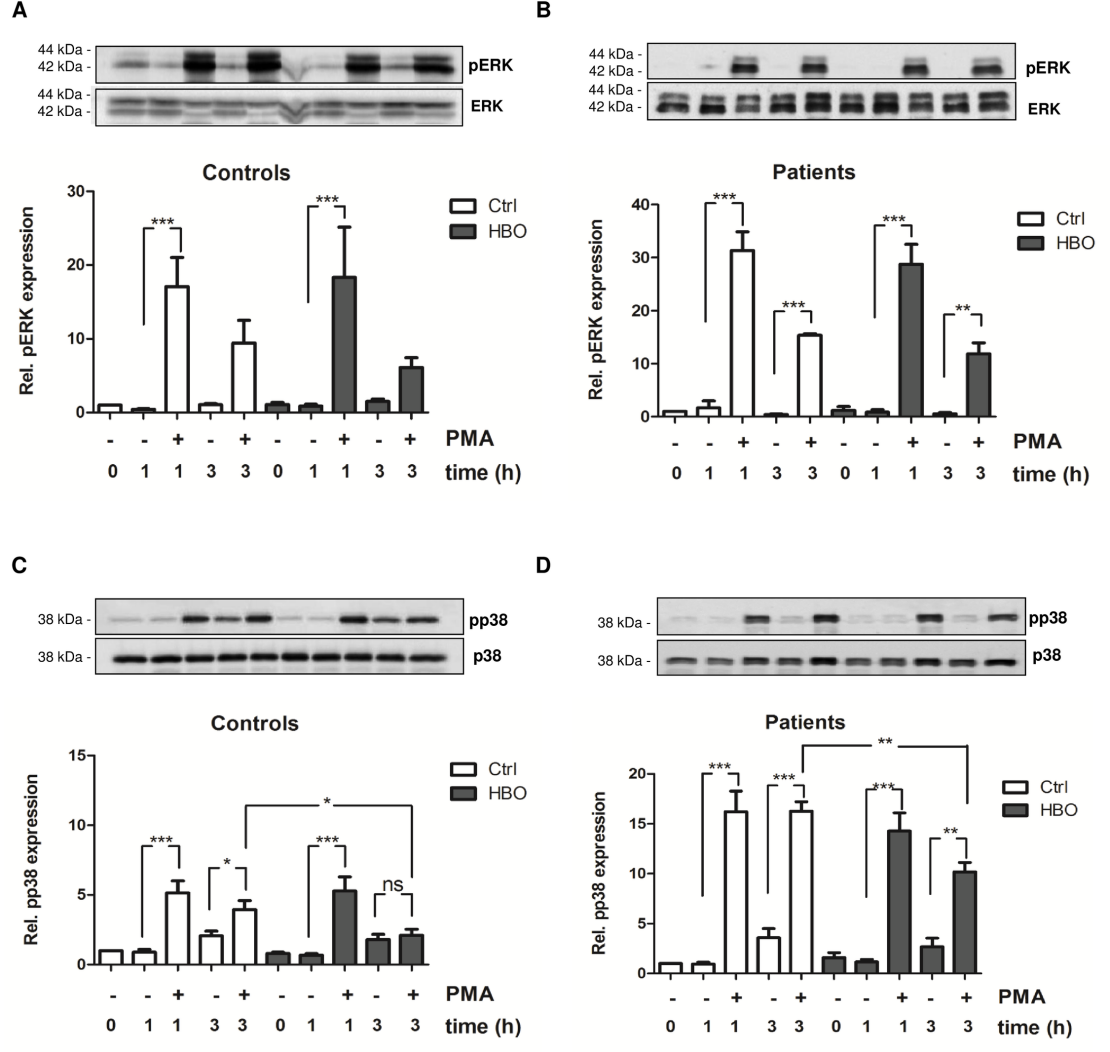
Effect of HBO on the activity of ERK and p38 MAPK. Neutrophils from volunteers (Controls) (A, C; n = 6) and polytraumatized patients (Patients) (B, D; n = 4) were exposed to HBO (2.0 atm; 120 min) and further stimulated with PMA (20 nM) for 1 h and 3 h. ERK phosphorylation (pERK; A, B) as well as the amount of phosphorylated p38 (pp38; B, D) were determined by western blot analyses. Relative expression of phosphorylated protein was quantified vs. unphosphorylated form. Data are expressed as fold changes vs. control (0 h Ctrl, set as 1). One representative western blot is depicted. *p<0.05; **p<0.01; ***p<0.001; ns = not significant

The activation of protein kinase C (PKC) by PMA has also been shown to be important for ROS production in neutrophils and appears to depend on phosphorylation of p38 MAPK and ERK, by a pathway that may suppress apoptosis to permit NETosis. Inhibition of the NADPH oxidase complex in neutrophils reduces ROS generation, p38 and ERK phosphorylation as well as NETs release [33]. To further strengthen our results showing that ROS production in neutrophils is markedly reduced by HBO therapy, the effects of HBO on the activity of ERK and p38 was examined. The time dependent phosphorylation of the kinases was monitored following PMA treatment. PMA stimulation strongly increased the expression of phosphorylated ERK protein (pERK) and phosphorylated p38 (pp38), and thus the activity of the kinases, in control and trauma neutrophils (Fig 4). However, ERK activity was maximal after 1 h of stimulation and further decreased after 3 h. At this time, the activity of ERK was found to be significantly increased only in trauma neutrophils (Fig 4B). HBO treatment did not lower levels of phosphorylated ERK protein in control and trauma neutrophils, although a slight downregulated 3 h after PMA stimulation could be observed (Fig 4A and 4B). Of note, a strong regulation of p38 activity by HBO was found. Similar to the findings concerning ERK activity, PMA-mediated p38 activation was more pronounced in trauma cells when compared to control neutrophils (Fig 4C and 4D). Again, kinase activity was markedly reduced after 3 h of stimulation in controls, but not in patient cells. Levels of phosphorylated p38 were significantly reduced at 3 h in cells exposed to HBO compared to control cells in both, neutrophils isolated from volunteers (Fig 4C) and trauma patients (Fig 4D) showing direct correlation with the decline in ROS production and NETs release (Figs 1 and 2).

### Effect of HBO on antiapoptotic Mcl-1 protein expression

**Fig 5:**
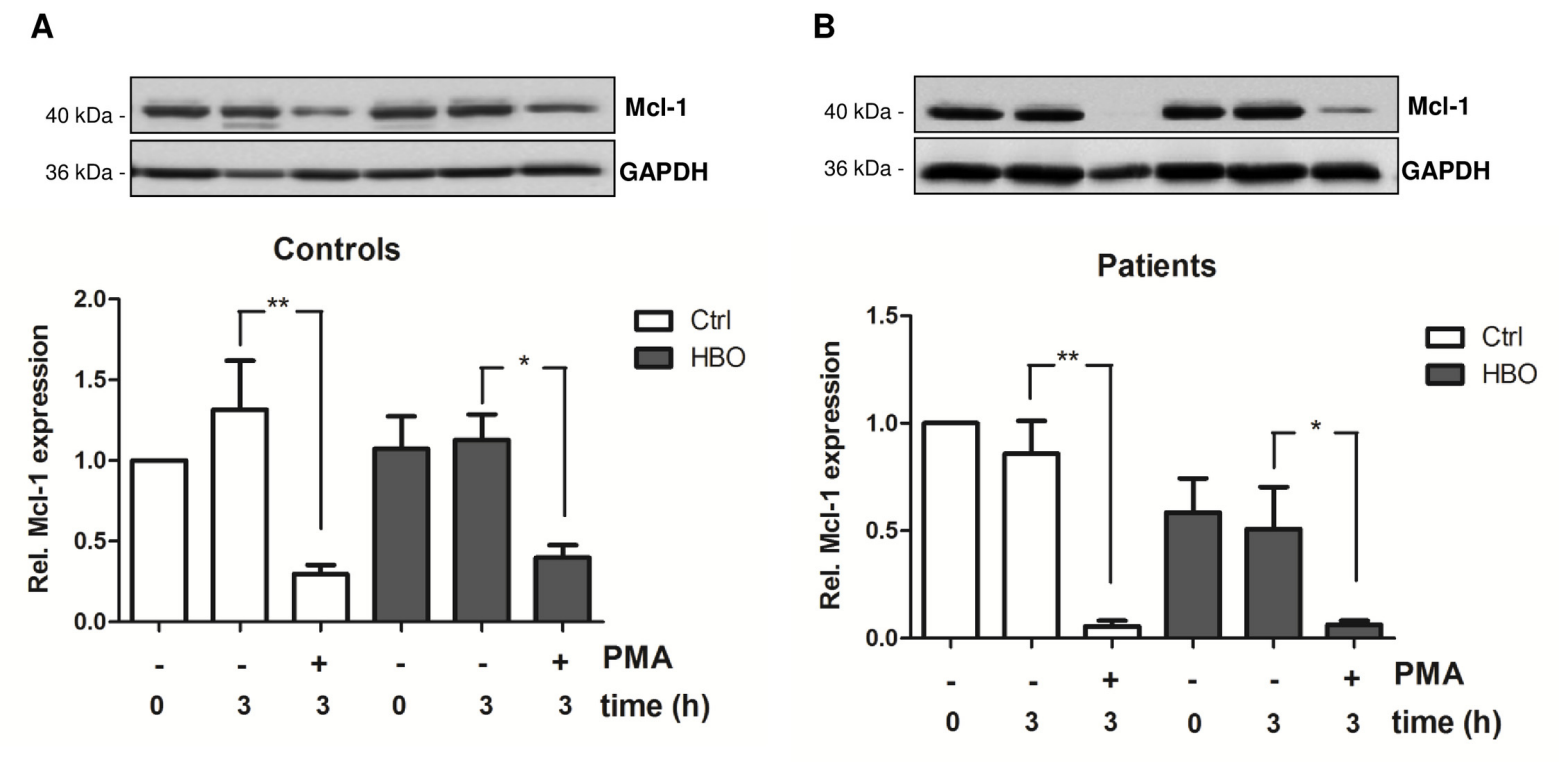
HBO does not alter the expression of antiapoptotic Mcl-1 protein. Neutrophils from healthy volunteers (Controls; n = 5) and polytraumatized patients (Patients; n = 4) were exposed to HBO (2.0 atm; 120 min) and further stimulated with PMA for 3 h. After that, Mcl-1 protein expression was quantified by western blot and normalized to GAPDH. Data are expressed as fold changes vs. control (0 h Ctrl, set as 1). *p<0.05; **p<0.01

Having shown, that HBO downregulates the activity of kinases and ROS production in activated patient cells as well as quiescent neutrophils, we asked if neutrophil apoptosis also becomes modulated by HBO therapy. The antiapoptotic Mcl-1 protein plays a critical role in the regulation of neutrophil apoptosis whereby decrease in intracellular protein levels usually correlate with increased apoptosis rate [34]. Previous studies postulated that HBO therapy provokes apoptosis in different cell types [35–37]. As depicted in Fig 5, stimulation of cells with PMA triggered a strong decrease in intracellular Mcl-1 protein levels already after 4 h. However, this finding is particularly interesting as PMA was also found to increase the activity of p38 and ERK, the last one promoting Mcl-1 stabilization [38]. Mcl-1 levels in control (Fig 5A) and trauma neutrophils (Fig 5B) were not altered by HBO. Accordingly, no significant change in apoptosis could be observed under our experimental setup (S1 Fig).

## Discussion

HBO is indicated for treating of decompression sickness, arterial gas embolism, and carbon monoxide poisoning [39], and evidence has accumulated that HBO therapy also has potent antiinflammatory effects [40]. In the current report, we demonstrate that HBO impedes the function of neutrophils isolated from polytraumatized patients by suppressing the production of ROS, downregulating the activity of MAPKs as well as NETs release.

In general, immunosuppressive effects of HBO have been excluded for long time and no effect on neutrophil viability or function was documented. However, this view changed over the past years. It has previously been demonstrated that exposure to HBO inhibits neutrophil ß2 integrin (CD11/CD18) function and adherence to the vascular endothelium, by increasing synthesis of ROS derived from inducible nitric oxide synthase (iNOS) and MPO and by nitric oxide-related protein modifications, in fact by S-nitrosation [41, 42]. These findings are in line with our own results showing that HBO does not affect the expression of the neutrophil adhesion molecules CD11b and CD66 (unpublished results). One interesting finding of this study was that HBO suppresses PMA-triggered NETosis. However, in neutrophils isolated from trauma patients, HBO-mediated reduction of NETs was visible but less pronounced when compared to cells isolated from healthy volunteers. Of note, trauma neutrophils displayed lower levels of NETs and ROS compared to controls. One explanation could be that NETs and ROS production strongly depend on PKC activity [43, 44], which becomes activated by PMA, but might be already activated in primed cells isolated from patients. However, although NETs levels did not decline significantly in trauma neutrophils exposed to HBO, a strong reduction of intracellular ROS production could be found, suggesting impairment of NADPH oxidase activity. Indeed, pre-treatment of neutrophils with the NADPH oxidase inhibitor DPI strongly reduced ROS production and NETs release [15]. After exposure to HBO, control neutrophils failed to adequately response to PMA stimulation as shown by inaccurate activation of the kinases ERK and p38 as well as ROS production. Both kinases, ERK and p38, were postulated to be involved in PMA-triggered NETosis. In this regard, Keshari *et al*. provided convincingly data demonstrating that PMA-induced ROS promote activation of ERK and p38 and subsequent NETs release from human neutrophils [33]. Although the authors also demonstrated that p38 acts upstream of ERK, we could not observe a time dependent activation. Both kinases were markedly upregulated by PMA after 1 hour whereby after 3 hours a progressive decrease in kinases phosphorylation could be determined, suggesting reduction in kinase activities. Of note, ROS-mediated ERK activation was largely restricted to ERK2, whereby ERK2 protein levels were apparently upregulated in patient cells. In this regard, ROS-induced ERK1/2 activation was already reported in a variety of cell types [45–47] and recent studies have identified functional differences in ERK1 and ERK2 [48]. Although it has been already demonstrated that ROS drive ERK2 translocation into the nucleus, where it directly binds to target proteins, a role in NETs formation remains debatable [49]. It has to be mentioned, that 1 hour and 3 hours usually represent quite late time points for the investigation of kinases activities and possible differences may appear already within few minutes. However, in this study, HBO-dependent diminution in neutrophil activity was seen later, namely after 3 hours. In this regard, we found exposure to HBO to not significantly impair PMA-mediated activation of ERK but to markedly reduce p38 activity at 3 hours after PMA stimulation in control and patient neutrophils. This observation is reflected by our finding showing strengthened suppression of ROS production 3 hours upon exposure to HBO.

NADPH-dependent ROS have also been reported to represent key modulators of neutrophils chemotaxis [32]. In our study, we did not find a reduction in chemotactic activity immediately after HBO exposure. Taking into account, that suppressive effects of HBO became evident temporary delayed, we cannot exclude, that HBO could result in compromised chemotactic migration.

To further elaborate the effect of HBO therapy on neutrophil apoptosis, levels of antiapoptotic Mcl-1 protein were quantified. Mcl-1 protein is known to have a very short half-life [50] and rapid degradation has been demonstrated by us and others in response to apoptotic stimuli [31, 51–53]. Notably, PMA stimulation of neutrophils resulted in a strong reduction of intracellular Mcl-1 protein levels, arguing for a negative regulation of Mcl-1 by PKC or downstream kinases. Indeed, it has been described that PKC is implicated in Mcl-1 degradation and inhibition of PKC activity resulted in protein stabilization [54]. Then again, ERK, which was upregulated by PMA, is known to stabilize the Mcl-1 protein [55]. Hakkim *et al*. reported that the Raf/MEK/ERK pathway upregulates Mcl-1 expression, thus inhibiting apoptosis to allow NETosis [56]. Contrary, it has recently been described that ROS inhibit AKT signaling and downregulate Mcl-1 protein [57]. On the basis of these observations, we speculate that PMA-triggered ROS production promotes fast Mcl-1 degradation, probably by the activation of the proteasomal pathway. This hypothesis is supported by a current report showing suppression of PMA-induced NETs formation by proteasome inhibitors [58]. In fact, HBO treatment did not significantly alter Mcl-1 protein levels after 3 h, neither in control nor in patient neutrophils. Accordingly, no differences in apoptosis rate were observed in cells exposed to HBO when compared to control cells suggesting that HBO does not affect apoptotic pathways.

Notably, it should be taken into account that our study lacks proper controls for the effects of pressure and hyperoxia alone. Second, neutrophils are known to infiltrate tissues where they come in close contact with other cells and inflammatory mediators, which in turn may alter their characteristics. Hence, here the wide variety of properties of neutrophil subtypes as well as of circulating, tissue neutrophils and those cells retaining to the circulation was not considered and may represent a further limitation of this study.

In summary, to our knowledge, this is the first report demonstrating that HBO therapy downregulates PMA-triggered ROS production, p38 activation, and thus ROS-dependent downstream pathways in neutrophils from severely injured patients. Our results implicate HBO therapy as a proper adjunctive therapy for the limitation of inflammation in critically ill patients. Nevertheless, the administration of HBO therapy remains controversial and risk versus benefit for any critical care disorder should be considered. Adverse effects of HBO therapy, although being rare, include damage to the ears, sinuses and lungs among others. Thus, hyperbaric chambers should be specifically designed for ICU patients allowing continuous patients’ treatment and ideally located near the ICU.

Future studies using animal models should verify the therapeutic potential and safe use of HBO for the treatment of patients suffering from systemic inflammation and prospective clinical use.

## Supporting Information

S1 Fig Effect of HBO on neutrophil apoptosis. Apoptosis was quantified in neutrophils isolated from healthy controls (n = 8) and polytraumatized patients (n = 4) 18 h after exposure to HBO.
(TIF)
