# Supplementary figures and images for: Hyperbaric Oxygen Reduces Production of Reactive Oxygen Species in Neutrophils from Polytraumatized Patients Yielding in the Inhibition of p38 MAP Kinase and Downstream Pathways
Source: PLoS One. 2016 Aug 16;11(8):e0161343. doi: 10.1371/journal.pone.0161343 (PMC4986935; doi:10.1371/journal.pone.0161343)

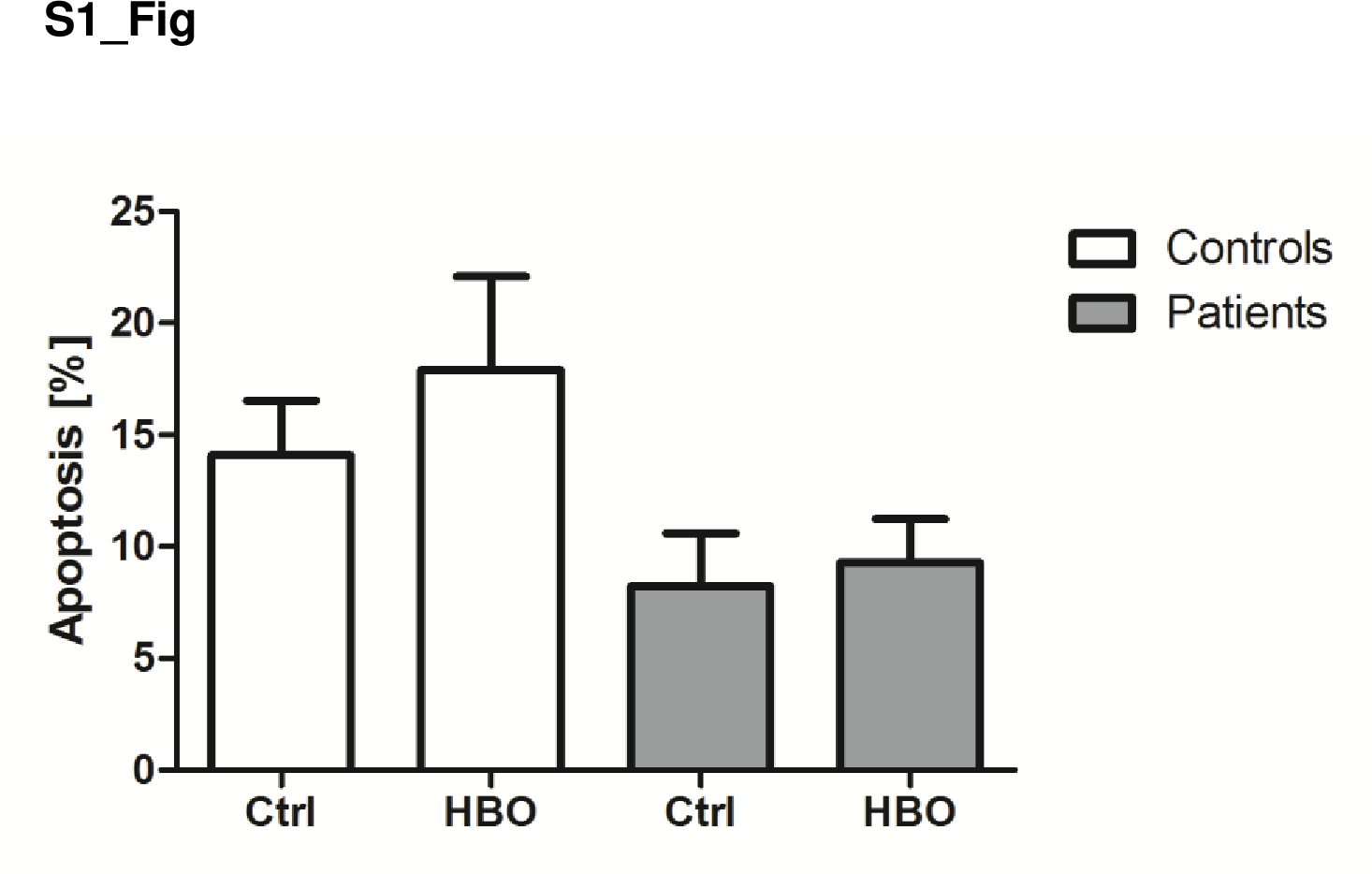

Supplement: S1 Fig — Apoptosis was quantified in neutrophils isolated from healthy controls (n = 8) and polytraumatized patients (n = 4) 18 h after exposure to HBO. (TIF) [file pone.0161343.s001.tif]
